# Risk-based screening and prognostic analysis for second primary malignancies in kidney cancer patients: a retrospective cohort study based on large-scale population and Mendelian randomization analysis

**DOI:** 10.7150/ijms.118457

**Published:** 2025-10-24

**Authors:** Mingrui Zou, Ruiyi Deng, Haode Liu, Jianhui Qiu, Peidong Tian, Jiaheng Shang, Jingcheng Zhou, Xueying Li, Lin Cai, Yizhou Wang, Kan Gong

**Affiliations:** 1 Department of Urology, Peking University First Hospital, Beijing, China.; 2 Institute of Urology, Peking University, Beijing, China.; 3 National Urological Cancer Center, Beijing, China.; 4 Department of Statistics, Peking University First Hospital, Beijing, China; 5 Department of Central Laboratory, Peking University First Hospital, Beijing, China.

**Keywords:** Kidney cancer, Mendelian randomization, Prognosis, Risk factors, Second primary malignancy

## Abstract

**Background**: Second primary malignancy (SPM) significantly impacts the survival of patients. This study endeavors to identify risk and prognostic factors of developing SPM after the first primary kidney cancer (FPKC), develop nomograms and explore potential mechanisms to optimize treatment strategies.

**Methods**: Data of patients diagnosed with FPKC between 2000 and 2020 were obtained from the SEER database. The standardized incidence ratio (SIR) was calculated to assess the relative risk of developing SPM in FPKC patients. Competing risk model as well as Cox regression analyses were employed to identify independent risk and prognostic factors, and nomograms were constructed and evaluated. Finally, to understand how FPKC influences the risk of developing SPM, we carried out Mendelian randomization (MR) and transcriptome-wide association study (TWAS) analyses.

**Results**: A total of 72408 and 5295 patients were included in stage I and II analysis, respectively. Risk distribution analysis revealed that FPKC patients exhibited a higher SPM risk than general population (SIR = 1.42, 95% CI: 1.40-1.44). Independent predictive factors were identified for model construction, and nomograms were developed. AUC of ROC, calibration curves and DCA illustrated excellent calibration and clinical applicability of the models. MR analyses indicated that kidney cancer might causally increase the risk of cancer in stomach, colon, rectum, lung, prostate, bladder, skin and eye. TWAS analysis identified 19 susceptibility genes associated with four types of cancers.

**Conclusion**: This study successfully established nomograms, delving into the potential mechanisms of developing SPM after FPKC. All these findings will promote the optimization of treatment strategies.

## Introduction

In recent years, with the advancement of cancer treatment techniques and the prolonged survival of patients, the prevalence of second primary malignancy (SPM) has been on the rise, emerging as a significant health issue among cancer survivors^1^. SPM is distinct from the initial metastasis or recurrence of a tumor; instead, it represents an independent malignant tumor that occurs after the cure or control of the initial malignancy^2^. Previous studies have found that kidney cancer survivors exhibited an elevated risk of developing SPMs, including prostate cancer, bladder cancer, colorectal cancer, lung cancer, melanoma, and non-Hodgkin lymphoma^3^, ^4^. A large-scale retrospective cohort study revealed that within 5 years, the cumulative incidence of SPM in kidney cancer patients reached 7.8%, which escalated to 12.3% within a decade^5^. Multiple factors contribute to the occurrence of these SPMs, including surveillance bias caused by the diagnosis of the first primary malignancy (FPM), treatment of FPM, common genetic factors associated with FPM and SPM, as well as their intricate interplays^6^-^8^. Furthermore, patients with SPM tend to have a poor prognosis. Wang et al. revealed that the 5-year overall survival (OS) of kidney cancer patients without SPM was 85.9%, while the 5-year OS of kidney cancer patients with SPM was 58.9%^9^. Therefore, understanding the risk and prognostic factors of developing SPM in kidney cancer patients is crucial.

Several studies have focused on the risk factors of developing SPM. For example, Zhan et al. reported that chemotherapy agents such as cisplatin and cyclophosphamide increased the risk of developing SPM in non-Hodgkin lymphoma patients (RR=2.10, 95% CI: 1.10-3.70)^10^. Jin et al. demonstrated that external beam radiotherapy (EBMT) was associated with increased incidence of SPM in thyroid cancer patients (HR=1.17, 95% CI: 1.03-1.33)^11^. Ma et al. elucidated that advanced age and small tumor size were risk factors of developing SPM in lung cancer patients^12^. However, limited research has focused on the risk and prognostic factors of developing SPM in first primary kidney cancer (FPKC) patients, and there is an urgent need for high-quality clinical cohorts and cutting-edge analytical strategies to unveil new insights into these factors of SPM in FPKC patients.

Nomograms are practical clinical prediction tools which are widely acknowledged for their extraordinary capacity in forecasting the risk and prognosis of various cancers^13^, ^14^. Epidemiological research strategies, such as Mendelian randomization (MR) and transcriptome-wide association study (TWAS) analysis, have been widely employed to investigate the causal associations between diseases as well as identify potential susceptibility genes across different tissues^15^, ^16^. Nevertheless, few studies have endeavored to develop nomograms to predict the occurrence and prognosis of SPM after kidney cancer. Moreover, there is a notable absence of studies employing MR analysis to explore the causal links between kidney cancer and SPM, alongside screening for susceptibility genes associated with SPM risk after kidney cancer.

**Figure 1:**
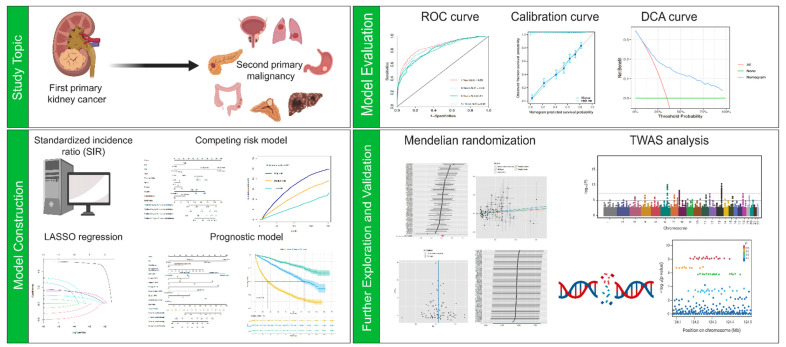
Study design.

In this study, we firstly calculated the standardized incidence ratio (SIR) and revealed that FPKC patients exhibited higher SPM risk than general population. Subsequently, we focused on the risk and prognostic factors of SPM after kidney cancer, and developed nomograms to predict the risk of developing SPM and prognosis of SPM after kidney cancer. Additionally, to investigate the causal associations between kidney cancer and SPM as well as how kidney cancer influences the risk of SPM, MR and TWAS analyses were performed. These findings will offer valuable insights into the monitoring and prevention of SPM in FPKC patients. The detailed study design was illustrated in **Figure 1**.

## Materials and Methods

### Data sources and patient selection

Data for FPKC patients were obtained from the Surveillance, Epidemiology, and End Results (SEER) database (https://seer.cancer.gov/) from 2000-2020 using SEER Stat 8.4.1. As data in the SEER database is publicly accessible, this study did not require informed consent and was exempt from the review of Internal Review Board (IRB).

According to the criteria of Warren and Gates from the National Cancer Institute, SPM was defined as metachronous invasive solid cancer developing ≥ 6 months after FPM^17^.

**Figure 2:**
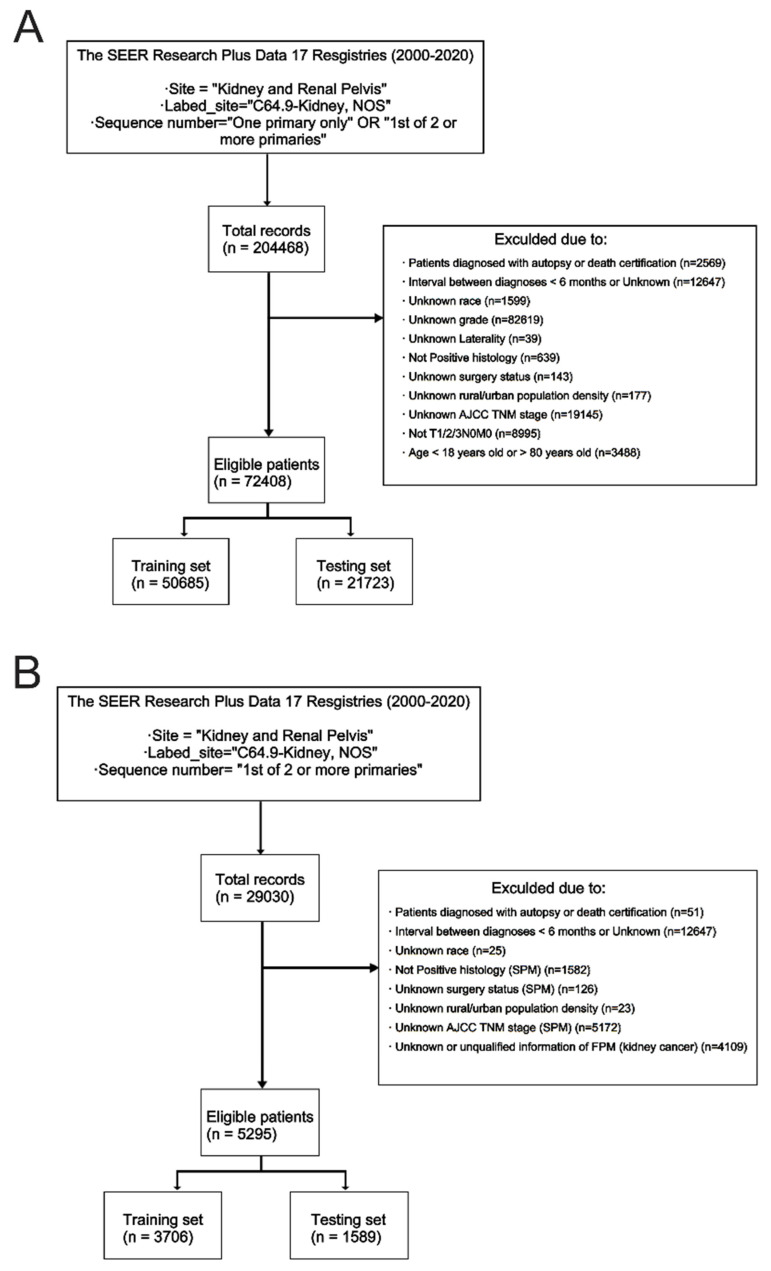
Study flowchart showing the process of constructing nomograms to predict the cumulative incidence (A) and prognosis of second primary malignancy (SPM) after kidney cancer (B).

For the formal analysis, the inclusion criteria were as follows: (1) Diagnosed age was between 18 and 80 years old; (2) Diagnosed histologically confirmed as FPKC; (3) The stage of kidney cancer was early or locally advanced (T1/2/3N0M0); (4) Detailed survival data and follow-up information should be provided. The exclusion criteria were: (1) Lack of histological conformation for diagnosis; (2) The type of reporting source was “Death certificate only” or “Autopsy only”; (3) Patients who had other malignancies prior to the diagnosis of primary kidney cancer; (4) Invasion of the regional structures listed in the T4 classification, invasion of lymph node (N1, N2, N3, or N_X_), or distant metastasis (M1); (5) Diagnosis interval between FPM and SPM was less than 6 months; (6) Incomplete information. After identification, the dataset was randomized 7:3 into the training set and testing set. The comprehensive screening process is presented in **Figure 2**. This study has been registered in ClinicalTrials.gov (NCT06531629).

### Clinical characteristics and outcome measurement

Baseline characteristics and clinicopathological data were gathered including age, sex, race, marital status, income, rural/urban population density, histologic type, grade, American Joint Committee on Cancer (AJCC) TNM stage, surgery history, tumor laterality, radiotherapy, chemotherapy, tumor size, the site of SPM, interval between the diagnosis of first primary malignancy (FPM) and SPM, as well as survival months and vital status. Some variables were categorized and regrouped, including Age at diagnosis (≤ 50 years, 50-60 years, 60-70 years, and 70-80 years), race (black, white and other), Marital status (No/Divorced/Widowed/Unknown and Yes), Income (≤ $75,000 and > $75,000), histologic type of kidney cancer (clear cell Renal Cell Carcinoma, ccRCC; Chromophobe Renal Cell Carcinoma, chRCC; papillary Renal Cell Carcinoma, pRCC; sarcomatoid Renal Cell Carcinoma, sRCC; and other), histologic grade (I, II, III/IV), Surgery (Not performed, Partial Nephrectomy (PN), Radical Nephrectomy (RN), local tumor destruction (LTD), and other), laterality (bilateral, left and right), site of SPM (urinary system, digestive system, reproductive system, respiratory system, and other), tumor size of FPM (≤ 2 cm, 2-3 cm, 3-4 cm, and > 4cm). The follow-up period was defined as the time from diagnosis to death, loss, to follow-up, or the end of study. The primary endpoint of stage I analysis of this study was the development of an SPM occurring more than 6 months after FPM. In the stage II analysis, overall survival (OS) served as the primary endpoint, which was defined as the time interval from the diagnosis of SPM to the death or the last follow-up for patients who did not die. The final follow-up data was December 31, 2020.

### Statistical analysis

#### Descriptive statical analysis

Statistical analyses were performed using R statistical software (Version 4.3.3). Categorical variables were depicted as frequencies (percentages), with comparisons between groups carried out using chi-square test. Continuous variables with normal distribution were represented as mean ± standard deviation (SD), and comparison between groups were executed using *t*-test. In case continuous variables exhibiting abnormal distribution, they were represented by median (Interquartile range (IQR)), and group comparisons were conducted using the Mann-Whitney U test. Two-sided *p* < 0.05 was considered statistically significant.

#### Risk distribution of SPM

To unveil the risk of specific types of SPM after kidney cancer, we calculated the SIR using the “MP-SIR section” of SEER Stat 8.4.1. SIR served as an estimate of relative risk, which was defined as the ratio of the observed number of patients with SPM to the expected number of patients to develop SPM based on the incidence of general population. Statistical results are shown in the presentation of SIR with 95 % confidence intervals (95 % CI).

#### Predictive factors identification and construction of nomograms

To investigate the risk factors for SPM after kidney cancer, we performed Fine-Gray competing risk regression analysis to assess the cumulative incidence of SPM^18^. This approach was specifically chosen because death from any cause prior to an SPM diagnosis acts as a significant competing event. The traditional Cox proportional hazards model, which treats deaths as non-informative censoring, would violate this assumption and potentially lead to an overestimation of the cumulative incidence of SPM. In contrast, the Fine-Gray model directly models the sub-distribution hazard, allowing for a more accurate and clinically meaningful estimation of the cumulative incidence function (CIF) in the presence of competing risks. Both univariable and multivariable analyses were conducted, and dying of all cases were considered competing events. Variables with *p* < 0.05 in the univariable analysis or deemed clinically significant would be included in the multivariable analysis. Variables with *p* < 0.05 in the multivariable analysis were identified as independent risk factors. Based on the identified risk factors, we constructed a nomogram to forecast the 3-, 5-, 7-, and 10-year risk of developing SPM. To translate the continuous total scores from the nomogram into clinically practical risk strata, patients were categorized into low-, medium-, and high-risk groups based on the tertiles of their scores. This data-driven approach was selected due to the absence of pre-established, clinically validated cut-off points for this specific outcome. Using tertiles ensures an objective and reproducible stratification, creating three equally sized groups for robust statistical comparison and facilitating a straightforward interpretation for clinical decision-making.

To further identify risk factors influencing the prognosis of FPKC patients with SPM, we conducted univariable and multivariable Cox proportional hazards regression analyses. Variables with *p* < 0.05 in the univariable analysis or deemed clinically significant would be included in the multivariable analysis. Variables with *p* < 0.05 in the multivariable analysis were identified as independent risk factors. Least absolute shrinkage and selection operator (LASSO) regression analysis was applied to further screen variables. Based on the identified variables with non-zero coefficients, the nomogram to predict the 1-, 3-, 5-, and 10-year possibility of survival was developed. Similarly, patients were categorized into low-, medium-, and high-risk groups based on the tertiles of their scores.

C-index, area under receiver operating characteristic (ROC) curve (AUC) and calibration curves were used to evaluate the discrimination capacity of the models and the consistency between actual result and predicted probability, respectively. Decision curve analysis (DCA) was used to assess the clinical efficacy of the prognostic nomogram.

### Mendelian randomization (MR) analysis

To further investigate the causal associations between kidney cancer and different types of SPM, we conducted two-sample MR analyses. MR is a well-known epidemiological strategy which is based on three hypotheses: (1) The genetic variants used as instrumental variables (IVs) should be solely related to the exposure; (2) The IVs are not allowed to be associated with any confounding factors; (3) The IVs are only allowed to exert an effect on the outcome via the exposure^16^.

In this study, kidney cancer served as the exposure and common SPM types of kidney cancer in clinical practice served as outcomes. Through MR analysis, we could find out whether FPKC would causally increase the risk of SPM. Based on previous studies and the results of SIR analysis, we selected 11 types of cancer as the outcomes of the MR analyses (gastric cancer, colorectal cancer, hepatocellular carcinoma, lung cancer, prostate cancer, bladder cancer, skin cancer, thyroid cancer, eye and adnexa cancer, adrenal gland cancer, and pancreatic cancer), as they were the most common SPM in patients with kidney cancer^19^, ^20^. The GWAS data of kidney cancer were collected from a large-scale genome-wide association study (GWAS)^21^, and GWAS data of other 11 types of cancers were obtained from IEU Open GWAS (https://gwas.mrcieu.ac.uk) and R10 release results of FinnGen consortium. The detailed information of these GWAS data is demonstrated in **Table S1**.

*P* < 5×10^-8^ was set as the genome-wide significant threshold to obtain single nucleotide polymorphisms (SNPs) strongly associated with kidney cancer. Subsequently, we clumped these SNPs (kb = 10,000, r^2^ = 0.001) to avoid linkage disequilibrium. In addition, SNPs with F-statistics less than 10 were regarded as weak IVs and were removed. Finally, palindromic SNPs and SNPs containing missing data were filtered. Furthermore, we searched the PhenoScanner database (http://www.phenoscanner.medschl.cam.ac.uk/phenoscanner) to investigate whether the selected IVs were associated with established risk factors of specific cancers. If such associations were found, the SNP was excluded^22^. In MR analysis, inverse-variance weighted (IVW) method served as the primary method to evaluate the causal association^23^. For IVW analysis, when there was no significant heterogeneity, fixed effect IVW model was employed; in case of significant heterogeneity, random effect model would be applied^23^, ^24^. Furthermore, other robust methods including MR-Egger regression, Weighted median and Weighted mode were also used, as they could provide reliable estimates of causal associations under wider conditions to mitigate the bias caused by ineffective IVs and horizontal pleiotropy^25^-^27^. Using the R package “TwoSampleMR” (Version 0.5.8), we conducted Steiger filtering analysis to evaluate whether the results were influenced by reverse causality. Reverse causality was considered absent if the direction is “TRUE” and the *p*-value < 0.05. In order to evaluate potential heterogeneity and horizontal pleiotropy, so as to verify the reliability of our results, we conducted sensitivity analyses. Cochran's Q test and funnel plot were used to evaluate the heterogeneity of the selected IVs (Heterogeneity is considered to exist when *p* < 0.05 or the funnel plot shows asymmetry)^24^. In addition, we used MR pleiotropy residual sum and outlier (MR-PRESSO) test to identify horizontal pleiotropy and potential pleiotropic outliers^28^. The MR Egger intercept test was also used to evaluate horizontal pleiotropy (Horizontal pleiotropy is considered to exist when *p* < 0.05)^25^. Leave-one-out analysis was conducted to assess the impact of excluding a single SNP on overall causal association^29^.

Recognizing that small case numbers in outcome GWAS can lead to biased estimates, we conducted a validation analysis for the three SPM types with fewer than 500 cases in the primary analysis (Hepatocellular carcinoma, eye and adnexa cancer, and adrenal gland cancer). We sourced alternative, independent GWAS summary statistics for these cancers (Supplementary Table S1) and repeated the MR analysis using the same instrumental variables and methods.

Statistical results are shown in the presentation of odds ratios (OR) with 95 % CI with a nominal significance threshold of *p* < 0.05. In addition, to obtain more rigorous and accurate results, we used the false discovery rate (FDR) method to correct all *p*-values^30^. In MR analysis, significance results are defined as: (1) the *p*-value of the IVW method corrected by the FDR method < 0.05. (2) Directionality of the MR-Egger method and Weighted median method is consistent with IVW method. If the *p*-value of the IVW method < 0.05, but the corrected *p*-value > 0.05, this result is suggestively significant. “TwoSample MR” package (Version 0.5.8), “MRPRESSO” package (Version 1.0) and “MendelianRandomization” package (Version 0.9.0) were used in R (Version 4.3.3) to conduct the MR analysis.

### Identification for susceptibility genes for SPM

To further investigate how FPKC influences SPM, we conducted transcriptome-wide association study (TWAS) analyses and summary-data-based Mendelian randomization (SMR) analyses to identify specific susceptibility genes for different types of SPM after kidney cancer. TWAS integrated expression quantitative trait loci (eQTL) data with GWAS summary statistics to identify novel gene-trait associations. In this study, Functional Summary-based Imputation (FUSION) software was employed to integrate GWAS data of cancers and eQTL data of healthy kidney cortex as well as clear cell renal cell carcinoma^15^, ^31^. The detailed information of these GWAS data and pre-computed predictive models is demonstrated in **Table S1**. FUSION developed multiple predictive models, including BLUP, BSLMM, Elastic Net, GBLUP, and LASSO, to assess the overall effect of SNPs on gene expression weights (The model exhibiting the highest predictive accuracy was employed for determining the gene weights). Subsequently, the genetic effects of cancers were integrated with gene weights to perform TWAS analyses. FDR correction was also conducted, with FDR < 0.05 regarded as statistically significant. In this way, we analyzed the associations between gene expression in kidney cortex and the onset of cancers in diverse sites of the body, and also scrutinizing susceptibility genes for SPM after kidney cancer.

Finally, to explore the causal associations between identified genes and SPM, SMR analyses were performed. The eQTL data of kidney cortex from GTEx v8 were used as exposures, and GWAS data of cancers were used as outcomes. Common (Minor allele frequency (MAF) > 0.01) cis-eQTLs exhibiting significant (*p* < 5×10^-8^) association with gene expression were selected as IVs. Using SMR software (V.1.3.1), SMR analyses were performed, with *p*-value < 0.05 considered as statistically significant^32^. Furthermore, heterogeneity in dependent instruments (HEIDI) test was conducted to evaluate heterogeneity (Whether the observed causal association was influenced by linkage scenario). *P-*value of HEIDI test > 0.05 indicated the absence of significant heterogeneity^32^. To further investigate shared genetic signals between causal susceptibility genes and cancers, we performed colocalization analyses, and the detailed methods were mentioned in a previous study^33^.

## Results

### Risk of type-specific SPM

**Figure 3:**
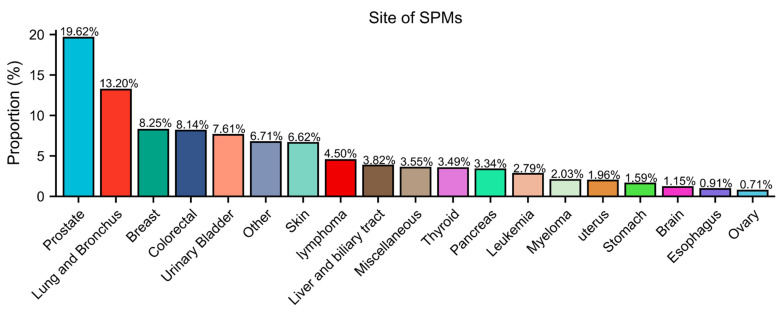
Sites of second primary malignancies (SPMs) after Kidney cancer.

The SIRs of SPM at different sites are demonstrated in **Table S2-S4**. Patients with FPKC were at higher risk of developing SPM as compared to general population (SIR = 1.42, 95% CI: 1.40-1.44). To be specific, the incidence of cancer at sites including thyroid (SIR = 3.63, 95% CI: 3.38-3.89), adrenal gland (SIR = 2.55, 95% CI: 1.36-4.36), liver (SIR = 1.54, 95% CI: 1.41-1.69), bladder (SIR = 1.51, 95% CI: 1.43-1.59), and prostate (SIR = 1.31, 95% CI: 1.27-1.35) were significantly higher than general population. The detailed results were presented in **Table S2-S4**. Furthermore, in **Figure 3**, we calculated the proportions of SPM across different body sites. The three most common sites for SPM were the prostate (19.62%), lung and bronchus (13.20%), and breast (8.25%).

### Demographic and clinicopathological characteristics

**Figure 2** depicted the process of patient selection. A total of 72408 FPKC patients were included in stage I analysis to investigate the risk factors of developing SPM, of whom 8583 (11.9%) patients developed SPM in the follow-up. The mean follow-up duration was 87.2 months. The detailed baseline demographic and clinicopathological characteristics of these patients in the training set (n = 50685) and testing set (n = 21723) are summarized in **Table S5**. Statistical analysis indicated that no significant differences existed between the two groups (*p* > 0.05).

For stage II analysis, a subgroup of 5295 FPKC patients with SPM was selected to construct a prognostic model aiming at predicting the OS of FPKC patients concurrently facing SPM. The mean interval between diagnoses of FPM and SPM was 56.54 months, and the mean follow-up duration was 53.54 months. The detailed baseline demographic and clinicopathological characteristics of these patients in the training set (n = 3706) and testing set (n = 1589) are summarized in **Table S6**. No statistically significant differences were detected between the two groups (*p* > 0.05).

### Stage I analyses (Competing risk model)

The univariable analyses demonstrated that age, sex, race, histologic type, grade, T stage, surgery, radiotherapy, and chemotherapy were potential predictive factors for developing SPM after kidney cancer (**Figure S1 and Table 1**). Subsequently, multivariable analyses identified age, sex, race, histologic type, grade, T stage, surgery, and chemotherapy as independent predictive factors (*p* < 0.05) for the development of SPM (**Table 1**). The hazard ratio (HR) and 95% CI of all variables in univariable and multivariable analyses are summarized in **Table 1**.

**Figure 4:**
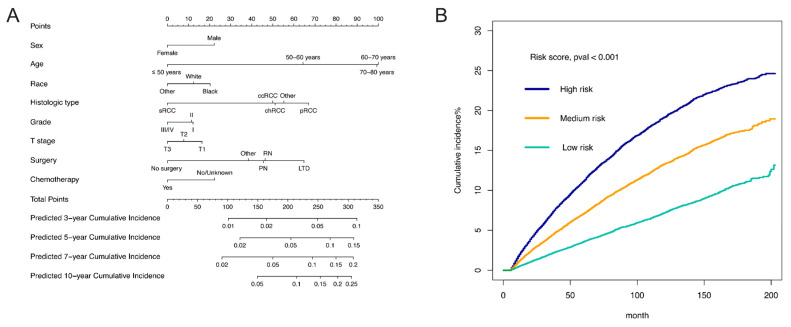
Assessment of the risk factors for second primary malignancy (SPM). (A) The nomogram. (B) The Cumulative incidence curves of patients at different risk of developing SPM.

Based on the eight identified independent risk factors, a nomogram was developed to display the 3-, 5-, 7-, and 10-year probabilities of developing SPM (**Figure 4A**). The scores of characteristics were calculated by the scale on the top, and the probabilities of developing SPM could be estimated by a perpendicular line from the total point axis to the axis corresponding to each time interval. We calculated the total points of each patient, designating the top third of patients with the highest scores as the high-risk group, the bottom third as the low-risk group, and those in the middle third as the medium-risk group. The two thresholds of points were 213 and 249. The nomogram could discriminate patients with different risks of developing SPM well (**Figure 4B**, *p* < 0.001). To be specific, the cumulative incidence of SPM in medium-risk patients was almost the same as that in all enrolled patients (Cumulative incidence of SPM in 100 months in medium-risk patients: 11.6%; Cumulative incidence of SPM in 100 months in all enrolled patients: 11.4%). Furthermore, the cumulative incidence of SPM in high-risk patients was significantly higher than that in all enrolled patients (Cumulative incidence of SPM in 100 months in high-risk patients: 16.8%), and the cumulative incidence of SPM in low-risk patients was significantly lower than that in all enrolled patients (Cumulative incidence of SPM in 100 months in low-risk patients: 6.0%) (**Figure 4B**). The C-index of the competing risk model was 0.620 (95% CI: 0.611-0.630) for the training set and 0.631 (95%CI: 0.617-0.647) for the testing set. The discrimination of the nomogram model was evaluated by the ROC curves and AUC values in the training set and testing set. As shown in **Figure S2**, the AUC of 3-, 5-, 7-, and 10-year ROC in the training set were 0.639, 0.636, 0.634, and 0.629, while the AUC of 3-, 5-, 7-, and 10-year ROC in the testing set were 0.652, 0.650, 0.641, and 0.635. The calibration curves of 3-, 5-, 7-, and 10-year of training and testing set are presented in **Figure S3** It appeared that the calibration curves were all very close to the ideal curves, indicating good consistency between the nomogram-predicted and the actual 3-, 5-, 7-, and 10-year incidence of SPM.

### Stage II analyses (Prognostic model)

To investigate the prognostic factors of FPKC patients with SPM, univariable and multivariable Cox regression analyses were conducted. In univariable analyses, 17 variables were significantly (*p* < 0.05) associated with OS of FPKC patients with SPM (**Table 2**). Subsequently, multivariable analyses identified age, marital status, income, interval between diagnoses, site of SPM, T stage of SPM, N stage of SPM, M stage of SPM, surgery status of SPM, histologic type of FPM, grade of FPM, radiotherapy of FPM, and chemotherapy of FPM as independent prognostic factors (*p* < 0.05) for the OS of FPKC patients with SPM (**Table 2**). The hazard ratio (HR) and 95% CI of all variables in univariable and multivariable analyses are summarized in **Table 2**. To further determine the variables ultimately included in the prognostic nomogram, LASSO Cox regression analysis was conducted to identify non-zero coefficients as potential OS risk factors (**Figure S4A and S4B**). Considering comprehensively the results of multivariate Cox regression analyses, LASSO regression analysis and clinical significance, age, marital status, interval between diagnoses, site of SPM, T stage of SPM, N stage of SPM, M stage of SPM, surgery status of SPM, grade of FPM, and chemotherapy of FPM were identified and incorporated into the predictive model.

**Figure 5:**
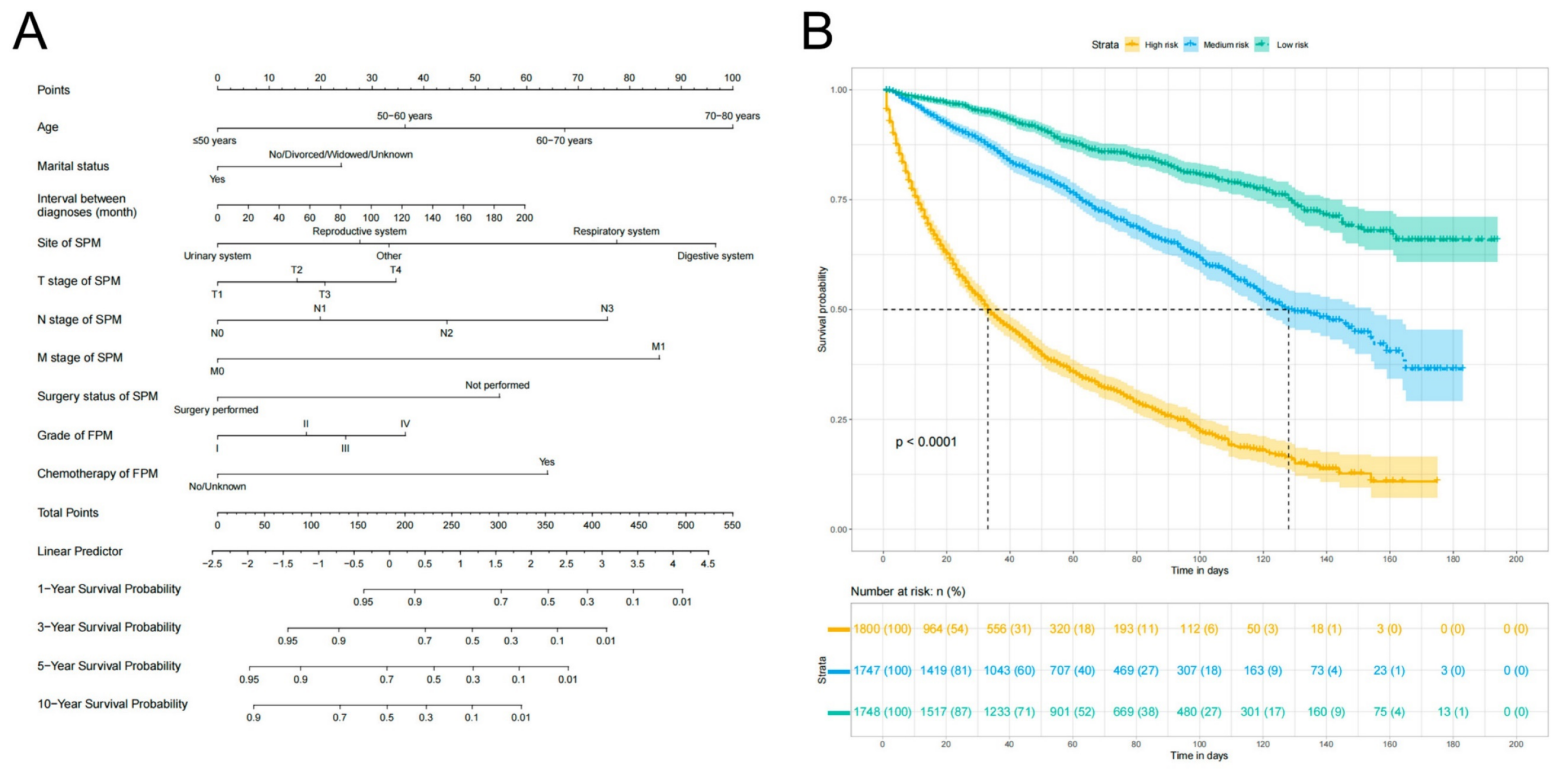
Assessment of overall survival (OS) of kidney cancer patients with second primary malignancy (SPM). (A) The Nomograms for predicting the 1-, 3-, 5-, and 10-year OS of kidney cancer patients with SPM. (B) Kaplan-Meier analysis for OS of kidney cancer patients with SPM at different risk.

**Figure 6:**
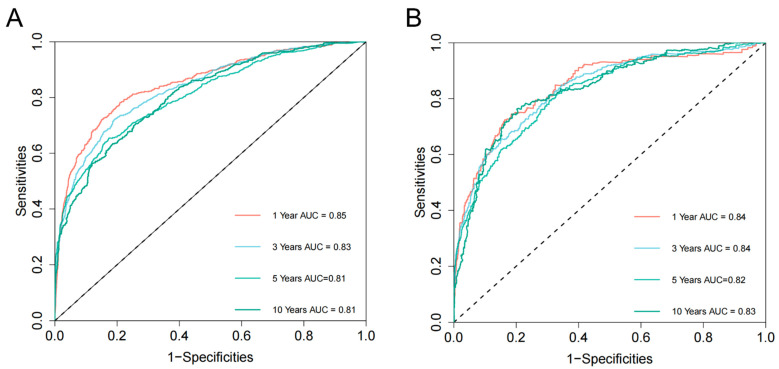
Nomogram receiver operating characteristic (ROC) curves and area under the curve (AUC) for the training set (A) and testing set (B).

Based on the 10 identified independent risk factors, we established a nomogram to predict the 1-, 3-, 5-, and 10-year OS of FPKC patients with SPM (**Figure 5A**). We calculated the total points of each patient, designating the top third of patients with the highest scores as the high-risk group, the bottom third as the low-risk group, and those in the middle third as the medium-risk group. The two thresholds of points were 142 and 199. The Kaplan-Meier survival curves demonstrated statistically significant differences among high-risk, medium-risk, and low-risk groups (**Figure 5B**, *p* < 0.001). To be specific, the median survival time of high-risk, medium-risk and low-risk groups were 33 months, 128 months and >200 months, respectively. The C-index of the prognostic model was 0.782 (95% CI: 0.769-0.796) for the training set and 0.784 (95%CI: 0.763-0.806) for the testing set. ROC curves and AUC values were employed to evaluate the discriminative power of the nomogram. In the training set, the AUC values for 1-, 3-, 5-, and 10-year OS prediction were 0.85, 0.83, 0.81, and 0.81 (**Figure 6A**). In the testing set, the AUC values for 1-, 3-, 5-, and 10-year OS prediction were 0.84, 0.84, 0.82, and 0.83 (**Figure 6B**). To evaluate the accuracy of our model, calibration curves were used to verify the consistency between our prognostic estimates and observed results. The calibration curves for 1-, 3-, 5-, and 10-year OS fit well with the 45° diagonal line in both the training set (**Figure S5A-S5D**) and testing set (**Figure S5E-S5H**), indicating an excellent performance of the nomogram. This nomogram has good predictive accuracy and reliability in forecasting the 1-, 3-, 5-, and 10-year OS of FPKC patients with SPM. DCA was applied to assess the clinical validity of the nomogram (**Figure S6**). The findings revealed that the nomogram model yielded substantial net benefit in both the training set and testing set, indicating that the nomogram exhibited robust predictive accuracy and clinical efficacy in predicting the OS of FPKC patients with SPM.

### Causal associations between kidney cancer and SPMs

**Figure 7:**
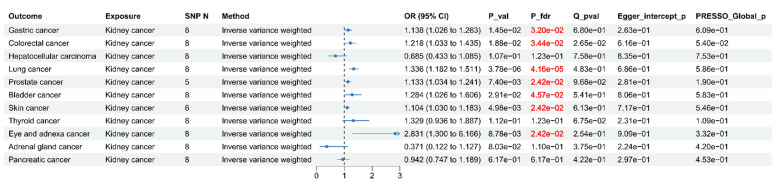
Forest plot for Mendelian randomization (MR) results between Kidney cancer and other cancer.

To further investigate the causal associations between kidney cancer and SPM, we conducted MR analyses. Eight SNPs were selected as IVs for kidney cancer (**Table S7**). As shown in **Figure 7**, after FDR correction, MR analyses revealed that kidney cancer might causally increase the risk of gastric cancer: (OR = 1.14, 95% CI: 1.03-1.26, *p* = 0.015), colorectal cancer: (OR = 1.22, 95% CI: 1.03-1.44, *p* = 0.019), lung cancer: (OR = 1.34, 95% CI: 1.18-1.51, *p* = 3.78×10^-6^), prostate cancer: (OR = 1.13, 95% CI: 1.03-1.24, *p* = 7.40×10^-3^), bladder cancer: (OR = 1.28, 95% CI: 1.03-1.61, *p* = 0.029), skin cancer: (OR = 1.10, 95% CI: 1.03-1.18, *p* = 4.98×10^-3^), and eye and adnexa cancer: (OR = 2.83, 95% CI: 1.30-6.17, *p* = 8.78×10^-3^). Cochran's Q test identified limited heterogeneity (*p* > 0.05). MR-PRESSO test and MR Egger intercept test failed to find any horizontal pleiotropy. Steiger filtering analysis demonstrated the absence of reverse causality (**Table S8**). The scatter plots, funnel plots, forest plots, and leave-one-out plots are presented in supplemental **Figure S7-S10**, which further enhance the reliability of our findings.

For cancers with low case number of GWAS (Hepatocellular carcinoma, eye and adnexa cancer, and adrenal gland cancer), we performed validation analyses. The association between kidney cancer and eye cancer was no longer significant (OR = 0.98, 95% CI: 0.49-1.94, *p* = 0.943), suggesting the initial finding was likely a false positive. No significant causal associations were observed for liver or adrenal cancers in either the primary or validation analyses (Table S9).

### Novel susceptibility genes associated with SPM risk after kidney cancer identified by TWAS analyses

Utilizing eQTL data from the healthy kidney cortex, single-tissue TWAS analysis (FUSION) identified 41 significant (FDR < 0.05) genes associated with the risk of prostate cancer, 3 for bladder cancer, 1 for colorectal cancer, 5 for gastric cancer, 2 for lung cancer, and 22 for skin cancer. Subsequently, utilizing eQTL data from renal clear cell carcinoma, single-tissue TWAS analysis (FUSION) identified 103 significant (FDR < 0.05) genes associated with the risk of prostate cancer, 2 for bladder cancer, 9 for gastric cancer, 15 for lung cancer, and 48 for skin cancer. The detailed results of TWAS analyses were presented in **Table S10 and S11**.

**Figure 8:**
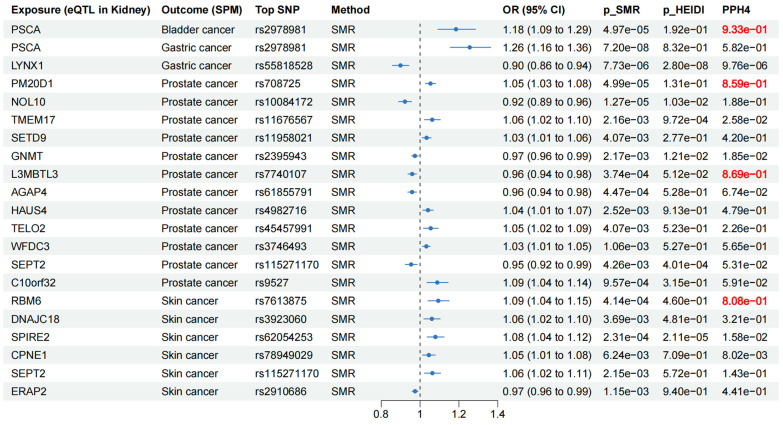
Forest plot for SMR results between eQTL in kidney and cancers.

**Figure 9:**
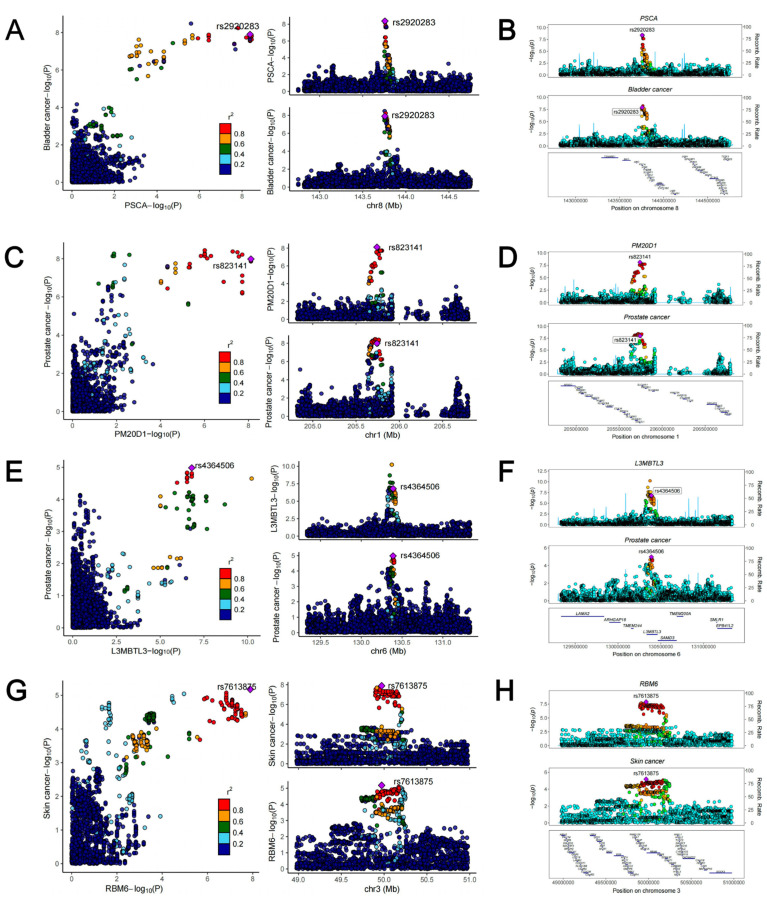
Regional association plots of colocalization analysis between PSCA, PM20D1, L3MBTL3 as well as RBM6 and cancers.

To further investigate the causal associations between identified susceptibility genes and SPM, we employed eQTL data of kidney cortex from GTEx and cancer GWAS data to perform SMR analyses. It is known that when using QTL data for MR analysis, SMR analysis could reach a higher statistical power. As shown in **Figure 8**, genetically predicted levels of PSCA in kidney were causally associated with increased risk of bladder cancer. Two genes (PSCA and LYNX1) presented causal associations with gastric cancer. 12 genes (PM20D1, NOL10, TMEM17, SETD9, GNMT, L3MBTL3, AGAP4, HAUS4, TELO2, WFDC3, SEPT2, and C10orf32) were causally associated with prostate cancer. Six genes (RBM6, DNAJC18, SPIRE2, CPNE1, SEPT2, and ERAP2) presented causal associations with skin cancer. Notably, PSCA could increase the risk of both bladder cancer (OR = 1.18, 95% CI: 1.09-1.29, *p* = 4.97×10^-5^) and gastric cancer (OR = 1.26, 95% CI: 1.16-1.36, *p* = 7.20×10^-8^), and it may be a core gene associated with the occurrence following kidney cancer diagnosis. HEIDI test found limited heterogeneity (**Figure 8**). Among the above causal associations, substantial colocalization evidence (PPH4 > 0.8) were found linking PSCA to bladder cancer, PM20D1 to prostate cancer, L3MBTL3 to prostate cancer, and RBM6 to skin cancer (**Figure 8 and Figure 9**). Detailed results of colocalization analyses were illustrated in **Table S12**.

## Discussion

Advanced cancer treatment techniques and the prolonged longevity of patients have contributed to the escalating prevalence of SPM. According to statistics from the National Cancer Institute, the incidence of multiple malignancies has doubled from 1979 to 2009, with one fifth of these cases arising in cancer survivors^34^, ^35^. A Danish cohort study demonstrated that compared with general population, patients with kidney cancer had a sustained 40% elevated long-term risk of SPM, which was mainly attributed to lung cancer (SIR = 2.4, 95% CI: 1.8-3.1) and bladder cancer (SIR = 4.6, 95% CI: 3.4-5.9)^36^. Another cohort study in Korea reported that the incidence of SPM was 13% higher in patients with kidney cancer than in the general population^37^. However, limited studies have delved into the risk factors associated with SPM after kidney cancer and prognostic factors for FPKC patients with SPM, highlighting the necessity for empirical evidence to optimize screening protocols and treatment strategies for SPM. In the present study, 8 variables including age, sex, race, histologic type, grade, T stage, surgery, and chemotherapy were identified as independent predictive factors for developing SPM after kidney cancer.

Age, marital status, interval between diagnoses, site of SPM, T stage of SPM, N stage of SPM, M stage of SPM, surgery status of SPM, grade of FPM, and chemotherapy of FPM were demonstrated to be independent prognostic factors for FPKC patients with SPM. All these factors were employed to construct nomograms to predict the probabilities of developing SPM and OS of FPKC patients with SPM. Results of C-index, ROC curves, calibration curves and DCA revealed that models exhibited excellent discrimination, calibration and clinical efficacy, underscoring their great potential as practical tools for optimizing screening protocols and treatment strategies tailored to SPM subsequent to kidney cancer. Furthermore, we performed MR, TWAS and colocalization analyses to clarify the causal associations between kidney cancer and other cancers, and identify the potential susceptibility genes through which kidney cancer influenced the risk of developing SPM.

MR analysis is a widely used epidemiological strategy to explore the causal associations between exposure and outcome. As it could mitigate confounding factors and reverse causal bias, we identified high-risk SPM types in FPKC patients. To be specific, kidney cancer might causally increase the risk of gastric cancer, colorectal cancer, lung cancer, prostate cancer, bladder cancer, and skin cancer, which was consistent with a previous study focusing on pan-cancer^38^. Hence, these 6 cancer types might be SPM types with the highest likelihood of occurrence in FPKC patients. In clinical practice, it is imperative to diligently surveil these 6 areas in FPKC patients to prevent the development of SPM. Through TWAS, SMR and colocalization analyses, we identified 19 reliable susceptibility genes in kidney associated with the development of SPM. Furthermore, we have noticed that PCSA in kidney could increase the risk of both bladder cancer and gastric cancer, and it exhibited significant colocalization with bladder cancer (PPH4 > 0.8). All these evidences indicated that PCSA might be a core gene in the development of SPM after kidney cancer. Prostate stem cell antigen (PSCA) is a cell surface protein that has different functions in different tissues^39^. Previous studies have demonstrated that several genetic variants of PSCA were associated with cancer susceptibility, such as rs2736098 for bladder cancer, and rs2294008 for gastric cancer^40^-^42^. Furthermore, PCSA was also reported to be up-regulated in renal cell carcinoma and bladder cancer, while to be down-regulated in gastric cancer^39^, ^42^, ^43^. However, most studies on the PSCA have focused on prostate cancer, and almost no studies investigated the links between PSCA levels in kidney and the risk of developing SPM. Consequently, understanding the specific mechanisms require further investigation. In the future, meticulous basic experiments may unveil this mystery.

To accurately predict the risk of developing SPM and the prognosis of these patients, we constructed and evaluated nomograms models. Here, we will further discuss the mechanisms through which identified independent factors played their roles. For demographic factors, increasing age, male sex, and black race were identified as independent risk factors for developing SPM. Elderly kidney cancer patients often exhibit diminished immune system functionality, compromised DNA repair capability and commonly have multiple chronic diseases, which put them at a high risk of developing SPM^44^. Previous studies have also shown that advanced age is also a risk factor for SPM in patients with other types of cancer^45^, ^46^. Risk difference brought by gender may be associated with differences in lifestyle, environment exposure and physiology between the sexes. To be specific, men are more likely to be exposed to tobacco and industrial pollutants, which are established risk factors for the onset of various cancers^47^. For races, black kidney cancer patients are at higher risk of developing SPM, this disparity could be attributed to genetic variations across races, socioeconomic status and access to healthcare. In contrast, black patients have relatively poor living conditions, and experience delays in cancer diagnosis and treatment, resulting in a higher risk of developing SPM^48^, ^49^.

We also found that patients with several histologic types of kidney cancer had higher risk of developing SPM, especially papillary renal cell carcinoma (pRCC) (HR = 1.19, 95% CI: 1.12-1.27). Thompson et al. demonstrated that compared to patients with clear cell renal cell carcinoma (ccRCC), patients with pRCC were more likely to develop SPM, especially prostate cancer (*p* = 0.003) and colon cancer (*p* = 0.041)^50^. A recent study suggested that clear cell papillary renal cell carcinoma (ccpRCC) patients might be significantly correlated with higher risk of developing SPM than ccRCC and pRCC patients^51^. ccpRCC was considered to share similar molecular and histological characteristics with both ccRCC and pRCC, which was reported to be associated with Fanconi anemia (VAFAs) pathway and presented distinct mutational characteristics from ccRCC and pRCC^51^. At present, the detailed mechanisms through which pRCC patients were at higher risk of developing SPM were still unclear, and further basic researches are needed to elucidate the intricate mechanisms. Furthermore, lower T stage and histological grade were considered risk factors for developing SPM after kidney cancer. Similar to being married and high income, patients with lower T stage and histological grade experience prolonged survival time, consequently exhibiting an increased likelihood of developing SPM during the survival period.

Two treatment strategies, surgery and chemotherapy, were considered to be independently associated with the risk of developing SPM. We noticed that compared to not performing surgery, Local tumor destruction (LTD) (HR = 1.94, 95% CI: 1.40-2.68), Radical Nephrectomy (RN) (HR = 1.61, 95% CI: 1.22-2.13) and Partial Nephrectomy (PN) (HR = 1.62, 95% CI: 1.22-2.14) could increase the risk of developing SPM. On the one hand, surgical procedures may inflict trauma on patients, leading to severe immune suppression and DNA impairment, consequently elevating the risk of SPM^52^, ^53^. On the other hand, surgery could alleviate the load of primary kidney cancer, thereby prolonging patients' survival. Owing to prolonged exposure to carcinogenic elements, the risk of developing SPM is higher. Chemotherapy diminishes the tumor burden in patients to a certain degree, consequently lowering the likelihood of developing SPM. However, as we know, prolonged exposure to high-dose chemotherapy drugs might potentially pose adverse effects on patients, underscoring the urgent need for high-quality clinical trials to investigate the optimal drugs and dosages of chemotherapy in kidney cancer patients, thereby mitigating the risk of developing SPM. Currently, there is a lack of relevant studies within the field of kidney cancer.

For prognostic nomograms, 10 independent predictive factors were identified. Advanced age and not being married were identified to be independently associated with poor prognosis in kidney cancer patients with SPM. As people grow older, their physiological and immune system functions gradually weaken, and some patients also suffer from chronic diseases, which are pivotal factors influencing their prognosis. Previous sociological studies have illustrated that married individuals typically have better health conditions and lower mortality rates^54^. To be specific, marriage plays a protective role by providing social support, improving lifestyle habits, and increasing economic resources^55^. Social support can alleviate stress and depression, and these negative emotional states have been proven to be associated with poor prognosis^56^.

In our study, we found that the prognosis of patients varied depending on the different locations of SPM. In general, patients with SPM located in the digestive and respiratory systems exhibited a poorer prognosis, while those with SPM located in the urinary and reproductive systems had a relatively more favorable prognosis. Given that kidney cancer belongs to the urogenital system, an area that has attracted attention and received treatment, SPM in the urogenital system can be detected earlier and receive targeted treatment to improve prognosis. Conversely, prioritizing the prevention and monitoring of tumors in urinary and reproductive systems might introduce a bias that overlooked the early detection of SPM occurred in other systems, allowing them to progress unchecked. It was widely recognized that tumors in digestive and respiratory systems advanced rapidly, exhibiting a higher degree of malignancy, ultimately leading to unfavorable prognosis for patients. Therefore, it was imperative to conduct further investigations on the prognosis of kidney cancer patients who developed SPM within specific organ systems or even a particular organ. However, relevant studies were scant, which is a topic worthy of further research.

We found that as the TNM stages of patients became higher, the prognosis was worse. Furthermore, the prognosis of patients who did not undergone surgery for SPM tended to be poorer. Interestingly, there was a trend toward shorter OS with an increase in the interval between diagnoses. Previous study found that shorter time interval to develop SPM might be associated with higher tumor burden, resulting in poorer prognosis^57^, ^58^. However, longer time interval implies ample time for kidney cancer to disseminate and progress, and the health status of the patients is not good. Subsequent occurrences of SPM further exacerbate this condition, leading to an unfavorable prognosis. Overall, further validation from additional cohorts is warranted to ascertain the impact of interval between diagnoses on the prognosis of kidney cancer patients with SPM.

Two characteristics of kidney cancer were also associated with the prognosis of patients. Patients with higher histological grade and taking chemotherapy for kidney cancer presented poor prognosis. As we have mentioned earlier, kidney cancer patients undergoing chemotherapy exhibited a lower probability of developing SPM. This may be attributed to the short survival period of patients after chemotherapy. As their survival time after chemotherapy is limited, the probability of developing SPM is also lower. Moreover, after developing SPM, the side effects of chemotherapy, such as damaging the immune system, inducing DNA damage and inhibiting DNA repair gradually influence the body, thereby impacting the prognosis of patients^59^. In the future, researchers could delve deeper into the effects of different doses of chemotherapy drugs on kidney cancer patients with SPM, thereby elucidating the implications of chemotherapy on patient prognosis.

This study possesses several noteworthy strengths. Firstly, to our best knowledge, this study is the first population-based study focusing on investigating the risk and prognostic factors of SPM after kidney cancer. Furthermore, we constructed and validated nomograms based on the identified factors, which demonstrated extraordinary performance in discrimination, calibration and clinical efficacy. These two nomograms were easy to use and could help physicians screen out patients at low or high risks of developing SPM and predict the OS of kidney cancer patients with SPM. In addition, given that the evidence level of observational study was relatively low, we conducted MR analysis to clarify the causal associations between kidney cancer and other types of cancer. The results of MR analysis indicated that gastric cancer, colorectal cancer, lung cancer, prostate cancer, bladder cancer, and skin cancer might be the SPM type with higher risk after kidney cancer as they presented causal links with kidney cancer. Finally, to deeper explain how kidney cancer increase the risk and prognosis of SPM, TWAS and SMR analyses were employed. We identified 19 promising susceptibility genes in kidney associated with the risk of developing SPM.

Nevertheless, some limitations should be acknowledged. Firstly, because of the nature of the SEER data, we were unable to adjust for some established cancer risk factors such as family history and lifestyle characteristics like tobacco use and alcohol consumption. Secondly, selection bias was inevitable given the observational retrospective nature of this study. Furthermore, we cannot determine the causal associations and specific mechanisms between these risk factors and the onset of SPM after kidney cancer. Thirdly, due to limited sample size, we were unable to focus on the onset of SPM in particular sites, such as second primary prostate cancer (SPPCa). However, the employment of MR analysis partially mitigated this deficiency by elucidating the causal associations between kidney cancer and other types of cancer. Fourthly, our genetic analyses, particularly the TWAS and SMR, were specifically designed to test the hypothesis that the elevated risk of SPM in FPKC patients is driven by dysregulated gene expression originating from the primary renal tissue. Consequently, we intentionally focused on data derived exclusively from kidney tissues. Regarding tissue specificity, our design is adept at identifying susceptibility genes whose pathogenic effects stem from renal expression. However, it is not designed to capture mechanisms where a shared genetic variant predisposes individuals to both FPKC and an SPM via gene expression primarily within the target tissue of SPM. Such extra-renal pathways represent an alternative hypothesis that could be explored in future multi-tissue studies. Fifthly, our analyses were restricted to cis-eQTLs from kidney tissue to ensure robust genetic instruments for causal inference. This standard, rigorous approach inherently limits our findings to genes under local genetic regulation, and we would not have detected causal genes whose expression is modulated by distal trans-eQTLs. Therefore, the genes identified herein should be considered strong, kidney-centric candidates for SPM risk, warranting further functional validation to elucidate their precise roles. At last, although the dataset was randomly split into training set and testing set for model construction and internal validation, additional external validation with other populations was still imperative. Given that the SEER database exclusively captures data from American population, and GWAS data as well as eQTL data used were from European countries, further investigation is warranted to ascertain the generalizability of our findings across diverse ethnicities and populations.

## Conclusion

In summary, age, sex, race, histologic type, grade, T stage, surgery, and chemotherapy were identified as independent risk factors of developing SPM in FPKC patients. Furthermore, age, marital status, interval between diagnoses, site of SPM, T stage of SPM, N stage of SPM, M stage of SPM, surgery status of SPM, grade of FPM, and chemotherapy of FPM were selected as independent prognostic factors for FPKC patients with SPM. Nomograms were established and validated to predict the probabilities of developing SPM and OS of FPKC patients with SPM. MR analyses unveiled the causal associations between kidney cancer and other 7 types of cancers (Gastric cancer, colorectal cancer, lung cancer, prostate cancer, bladder cancer, skin cancer, eye and adnexa cancer). TWAS and SMR analyses identified 19 causal susceptibility genes in kidney associated with the risk of developing SPM. All these findings might help physicians to formulate tailored screening protocols for SPM in individuals with kidney cancer and customized treatment regimens for FPKC patients with SPM.

## Supplementary Material

Supplementary figures and tables 5-6.

Supplementary tables 1-4, 7-12.

## Figures and Tables

**Table 1:** Univariable and multivariable analysis of competing risk models.

	Univariate analysis			Multivariate analysis		
	HR	95% CI	P	HR	95% CI	P
Age						
≤ 50 years	Reference			Reference		
50-60 years	2.01	1.86-2.16	< 0.001	2.01	1.87-2.17	< 0.001
60-70 years	2.91	2.71-3.12	< 0.001	2.95	2.74-3.17	< 0.001
70-80 years	2.88	2.66-3.11	< 0.001	2.99	2.76-3.23	< 0.001
Sex						
Female	Reference			Reference		
Male	1.27	1.21-1.32	< 0.001	1.30	1.24-1.36	< 0.001
Race						
Black	Reference			Reference		
White	0.95	0.89-1.02	0.140	0.94	0.88-1.01	0.079
Other	0.79	0.71-0.88	< 0.001	0.81	0.72-0.90	< 0.001
Rural/urban population density						
Counties in metropolitan areas of over 1 million population	Reference					
Counties in metropolitan areas of 0 to 1 million population	1.00	0.96-1.05	0.850			
Nonmetropolitan counties	1.03	0.97-1.10	0.330			
Histologic type						
ccRCC	Reference			Reference		
chRCC	0.93	0.94-1.04	0.190	0.99	0.90-1.10	0.900
Other	1.07	1.02-1.13	0.010	1.06	1.01-1.12	0.025
pRCC	1.33	1.25-1.41	< 0.001	1.20	1.12-1.28	< 0.001
sRCC	0.59	0.40-0.88	0.009	0.65	0.44-0.96	0.031
Grade						
I	Reference			Reference		
II	1.01	0.95-1.08	0.700	0.99	0.93-1.06	0.780
III/IV	0.94	0.87-1.00	0.051	0.90	0.84-0.97	0.004
T stage						
T1	Reference			Reference		
T2	0.88	0.82-0.95	< 0.001	0.91	0.85-0.98	0.011
T3	0.87	0.82-0.93	< 0.001	0.84	0.79-0.90	< 0.001
Surgery						
Not performed	Reference			Reference		
PN	1.41	1.07-1.86	0.015	1.64	1.24-2.17	< 0.001
RN	1.39	1.05-1.83	0.020	1.63	1.24-2.16	< 0.001
LTD	2.04	1.48-2.81	< 0.001	1.96	1.42-2.70	< 0.001
Other	1.52	1.12-2.07	0.008	1.56	1.14-2.13	0.005
Laterality						
Bilateral	Reference					
Left	1.18	0.45-3.10	0.730			
Right	1.18	0.45-3.08	0.740			
Radiotherapy						
No/Unknown	Reference			Reference		
Yes	0.71	0.47-1.07	0.100	0.84	0.55-1.27	0.41
Chemotherapy						
No/Unknown	Reference			Reference		
Yes	0.61	0.48-0.77	< 0.001	0.72	0.56-0.92	0.009

Abbreviations: HR, Hazard Ratio; CI, confidence interval; ccRCC, clear cell renal cell carcinoma; chRCC, Chromophobe cell renal cell carcinoma; pRCC, Papillary renal cell carcinoma; sRCC, Sarcomatoid renal cell carcinoma.

**Table 2:** Univariable and multivariable Cox proportional analysis of the associated factors for the OS of kidney cancer patients with SPM.

	Univariate analysis			Multivariate analysis		
	HR	95% CI	P	HR	95% CI	P
Age						
≤ 50 years	Reference			Reference		
50-60 years	1.35	1.09-1.66	0.006	1.47	1.19-1.82	< 0.001
60-70 years	2.04	1.67-2.49	< 0.001	2.26	1.86-2.79	< 0.001
70-80 years	3.36	2.75-4.11	< 0.001	3.56	2.89-4.38	< 0.001
Sex						
Female	Reference					
Male	1.08	0.98-1.19	0.121			
Race						
Black	Reference					
White	1.08	0.93-1.25	0.331			
Other	1.06	0.83-1.35	0.630			
Marital status						
No/Divorced/Widowed/Unknown	Reference			Reference		
Yes	0.74	0.67-0.81	< 0.001	0.75	0.69-0.83	< 0.001
Income						
≤ $75,000	Reference			Reference		
> $75,000	0.87	0.80-0.97	0.008	0.89	0.80-0.99	0.029
Rural/urban population density						
Counties in metropolitan areas of over 1 million population	Reference			Reference		
Counties in metropolitan areas of 0 to 1 million population	1.10	0.99-1.22	0.068	1.08	0.97-1.20	0.145
Nonmetropolitan counties	1.26	1.10-1.44	< 0.001	1.08	0.94-1.25	0.277
Interval between diagnoses						
Interval	1.002	1.001-1.003	< 0.001	1.004	1.002-1.005	< 0.001
Site of SPM						
Urinary system	Reference			Reference		
Digestive system	3.49	3.07-3.98	< 0.001	3.76	3.20-4.43	< 0.001
Reproductive system	1.16	0.99-1.37	0.066	1.68	1.39-2.03	< 0.001
Respiratory system	4.49	3.93-5.11	< 0.001	3.04	2.60-3.56	< 0.001
Other	1.13	0.96-1.34	0.149	1.71	1.42-2.06	< 0.001
T stage of SPM						
T1	Reference			Reference		
T2	1.20	1.07-1.34	0.002	1.29	1.15-1.44	< 0.001
T3	1.86	1.64-2.12	< 0.001	1.30	1.12-1.51	< 0.001
T4	4.98	4.31-5.75	< 0.001	1.72	1.46-2.03	< 0.001
N stage of SPM						
N0	Reference			Reference		
N1	1.91	1.68-2.18	< 0.001	1.26	1.08-1.46	0.003
N2	4.15	3.60-4.78	< 0.001	1.56	1.31-1.86	< 0.001
N3	6.94	5.50-8.76	< 0.001	2.29	1.76-2.99	< 0.001
M stage of SPM						
M0	Reference			Reference		
M1	7.67	6.80-8.65	< 0.001	2.92	2.52-3.39	< 0.001
Surgery status of SPM						
Not performed	Reference			Reference		
Surgery performed	0.50	0.46-0.55	< 0.001	0.46	0.41-0.52	< 0.001
Radiotherapy of SPM						
No/Unknown	Reference					
Yes	1.02	0.93-1.12	0.689			
Chemotherapy of SPM						
No/Unknown	Reference			Reference		
Yes	2.63	2.39-2.91	< 0.001	1.07	0.94-1.22	0.320
Histologic type of FPM						
ccRCC	Reference			Reference		
chRCC	0.80	0.63-1.03	0.084	0.86	0.67-1.11	0.240
Other	1.10	0.99-1.24	0.087	1.08	0.97-1.22	0.165
pRCC	0.94	0.82-1.07	0.337	0.91	0.79-1.04	0.167
sRCC	2.12	1.10-4.09	0.025	2.09	1.06-4.09	0.032
Grade of FPM						
I	Reference			Reference		
II	1.06	0.92-1.22	0.407	1.16	1.01-1.34	0.041
III	1.20	1.03-1.40	0.017	1.24	1.06-1.46	0.008
IV	1.51	1.19-1.91	< 0.001	1.31	1.02-1.69	0.033
Laterality of FPM						
Bilateral	Reference					
Left	0.46	0.06-3.25	0.434			
Right	0.46	0.07-3.30	0.444			
Surgery status of FPM						
Not performed	Reference			Reference		
Surgery performed	0.40	0.24-0.66	< 0.001	0.89	0.52-1.53	0.674
Radiotherapy of FPM						
No/Unknown	Reference			Reference		
Yes	2.82	1.77-4.49	< 0.001	2.20	1.35-3.56	0.001
Chemotherapy of FPM						
No/Unknown	Reference			Reference		
Yes	2.25	1.66-3.06	< 0.001	2.00	1.45-2.76	< 0.001
Size of FPM						
≤ 2 cm	Reference			Reference		
2-3 cm	1.16	0.97-1.39	0.103	1.07	0.89-1.28	0.463
3-4 cm	1.24	1.03-1.48	0.020	1.09	0.90-1.30	0.380
> 4cm	1.42	1.21-1.66	< 0.001	1.17	0.99-1.38	0.060

Abbreviations: HR, Hazard Ratio; CI, confidence interval; ccRCC, clear cell renal cell carcinoma; chRCC, Chromophobe cell renal cell carcinoma; pRCC, Papillary renal cell carcinoma; sRCC, Sarcomatoid renal cell carcinoma.

## Abbreviations

SPM: Second primary malignancy
FPKC: First primary kidney cancer
SEER: Surveillance, Epidemiology, and End Results
SIR: Standardized incidence ratio
MR: Mendelian randomization
TWAS: Transcriptome-wide association study
AUC: Area under curve
ROC: Receiver operating characteristic
DCA: Decision curve analysis
FPM: First primary malignancy
EBMT: External beam radiotherapy
IRB: Institutional Review Board
AJCC: American Joint Committee on Cancer
PN: Partial Nephrectomy
RN: Radical Nephrectomy
LTD: Local tumor destruction
OS: Overall survival
SD: Standard deviation
IQR: Interquartile range
CI: Confidence interval
LASSO: Least absolute shrinkage and selection operator
IVs: Instrumental variables
GWAS: Genome-wide association study
SNPs: Single nucleotide polymorphisms
IVW: Inverse-variance weighted
MR-PRESSO: MR pleiotropy residual sum and outlier
OR: odds ratio
FDR: False discovery rate
eQTL: Expression quantitative trait loci
FUSION: Functional Summary-based Imputation
MAF: Minor allele frequency
HEIDI: Heterogeneity in dependent instruments
HR: Hazard Rati
ccRCC: Clear cell renal cell carcinoma
chRCC: Chromophobe cell renal cell carcinoma
pRCC: Papillary renal cell carcinoma
sRCC: Sarcomatoid renal cell carcinoma
PSCA: Prostate stem cell antigen
