# Supplementary material for: Risk-based screening and prognostic analysis for second primary malignancies in kidney cancer patients: a retrospective cohort study based on large-scale population and Mendelian randomization analysis
Source: Int J Med Sci. 2025 Oct 24;22(16):4432–50. doi: 10.7150/ijms.118457 (PMC12595329; doi:10.7150/ijms.118457)
Supplement: Supplementary file 1 — Supplementary figures and tables 5-6. [file ijmsv22p4432s1.pdf]

Risk-based screen and prognostic analysis for second primary malignancies in kidney cancer patients: A retrospective cohort study based on large-scale population and mendelian randomization analysis

Supplementary Figures

Author names and affiliations: Mingrui Zou [1,2,3,†], Ruiyi Deng [1,2,3,†], Haode Liu [1,2,3,†], Jianhui Qiu [1,2,3], Peidong Tian [1,2,3], Jiaheng Shang [1,2,3],Jingcheng Zhou [1,2,3], Xueying Li[4], Lin Cai [1,2,3], Yizhou Wang [5,*] and Kan Gong [1,2,3,*]

[1]Department of Urology, Peking University First Hospital, Beijing, China.

[2]Institute of Urology, Peking University, Beijing, China.

[3]National Urological Cancer Center, Beijing, China.

[4]Department of Statistics, Peking University First Hospital, Beijing, China

[5]Department of Central Laboratory, Peking University First Hospital, Beijing, China.

[†]These authors contributed equally to this work and should be considered co-first authors.

* ***Corresponding authors***: Kan Gong(kan.gong@bjmu.edu.cn) AND Yizhou Wang (yizhouwang@bjmu.edu.cn)

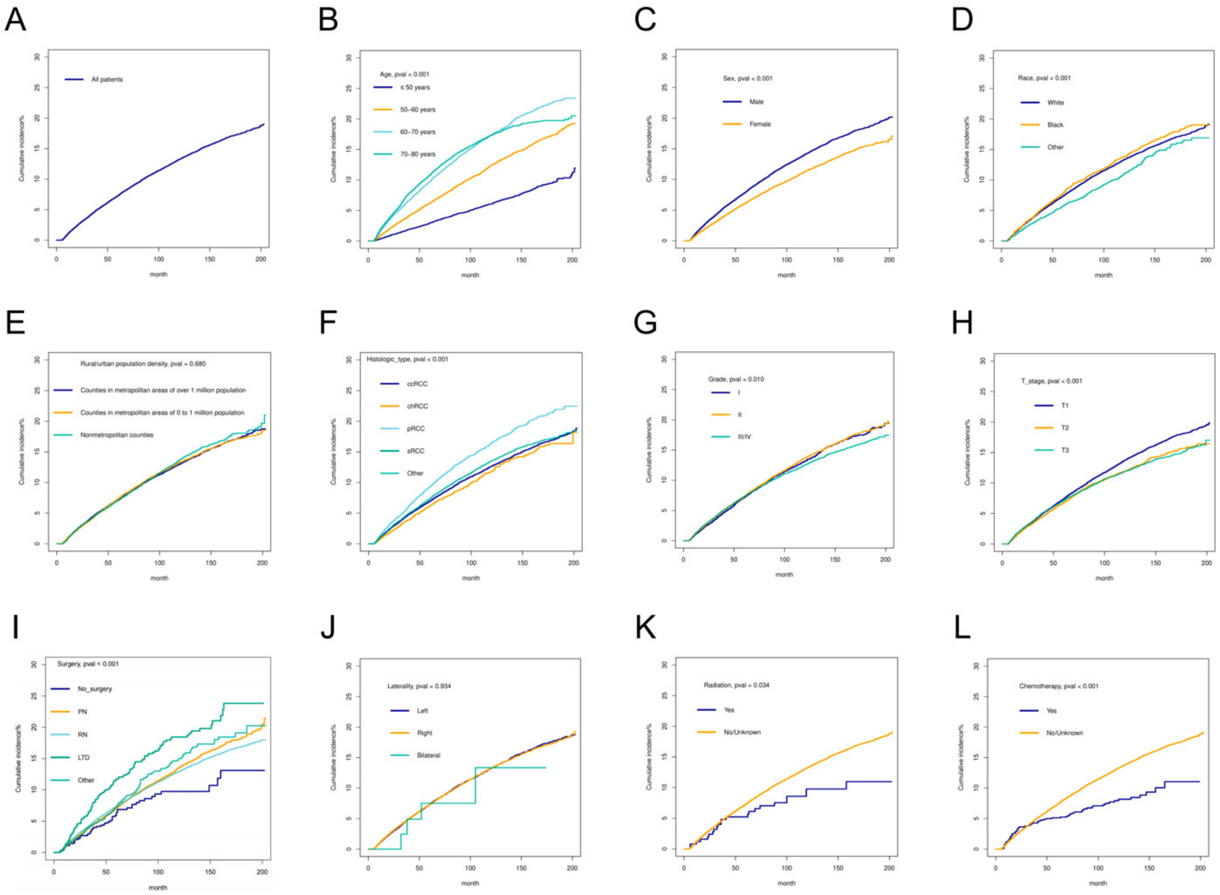

Figure S1. Curves of the cumulative incidence function in the competing risk univariable analysis of the incidence of second primary malignancy (SPM) after kidney cancer.

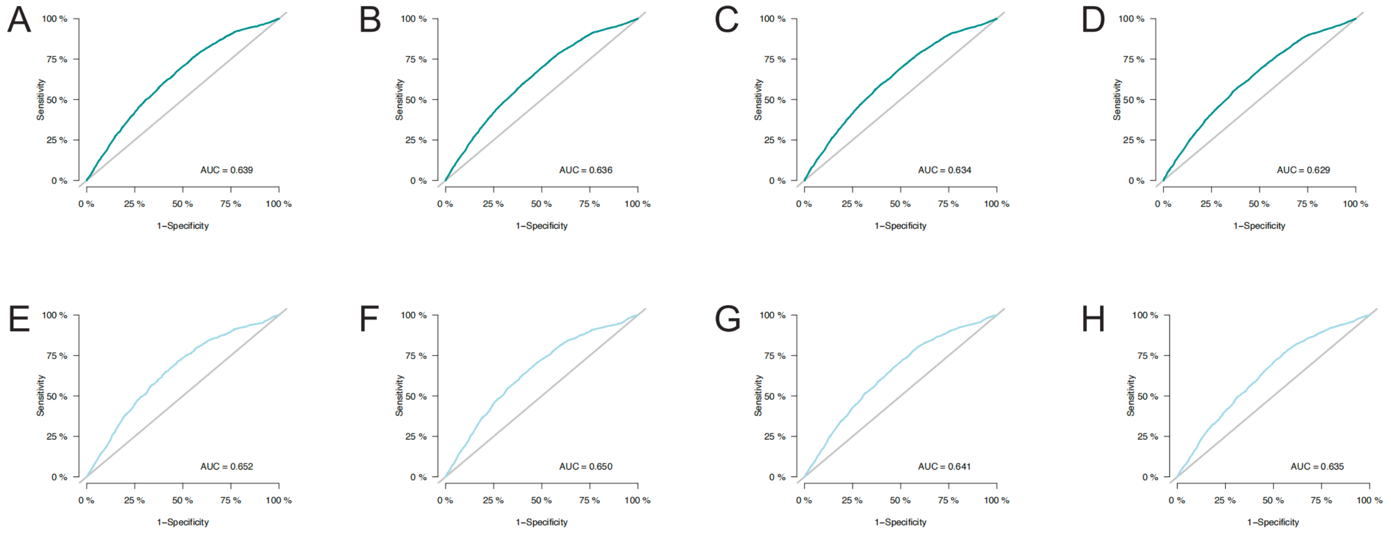

Figure S2. (A-D) The receiver operating characteristic (ROC) curve and the area under the curve (AUC) of the developed model for predicting 3-, 5-, 7- and 10-year risk of developing second primary malignancy (SPM) risk in the training set. (E-H) The receiver operating characteristic (ROC) curve and the area under the curve (AUC) of the developed model for predicting 3-, 5-, 7- and 10-year risk of developing second primary malignancy (SPM) risk in the testing set.

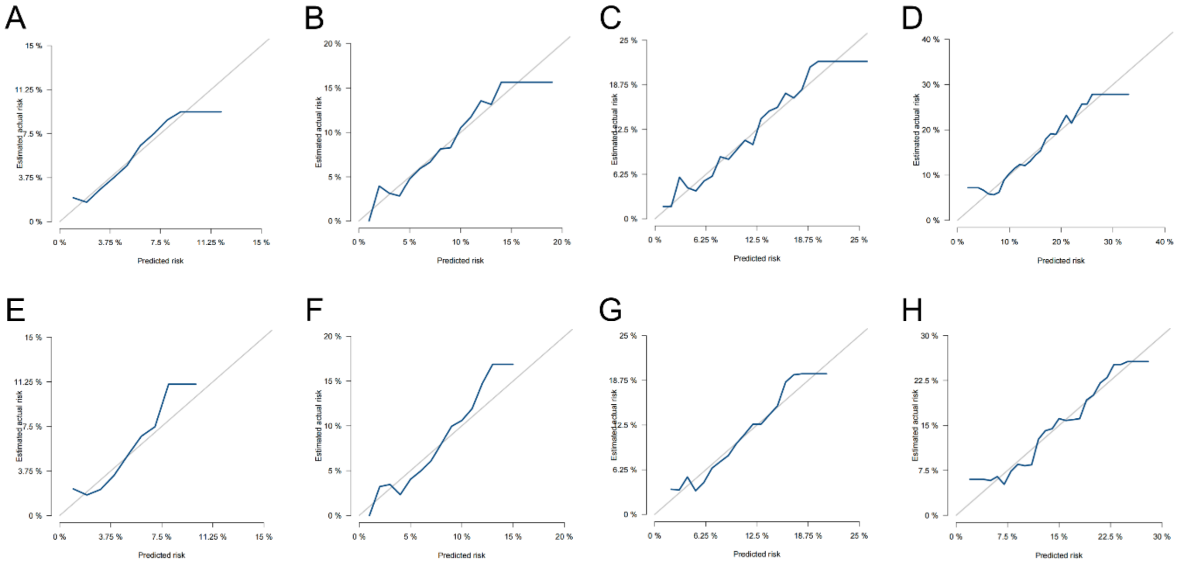

Figure S3. (A-D) The calibration curves of nomogram for predicting 3-, 5-, 7- and 10-year probabilities of SPM in the training set. (E-H) The calibration curves of nomogram for predicting 3-, 5-, 7- and 10-year probabilities of SPM in the testing set.

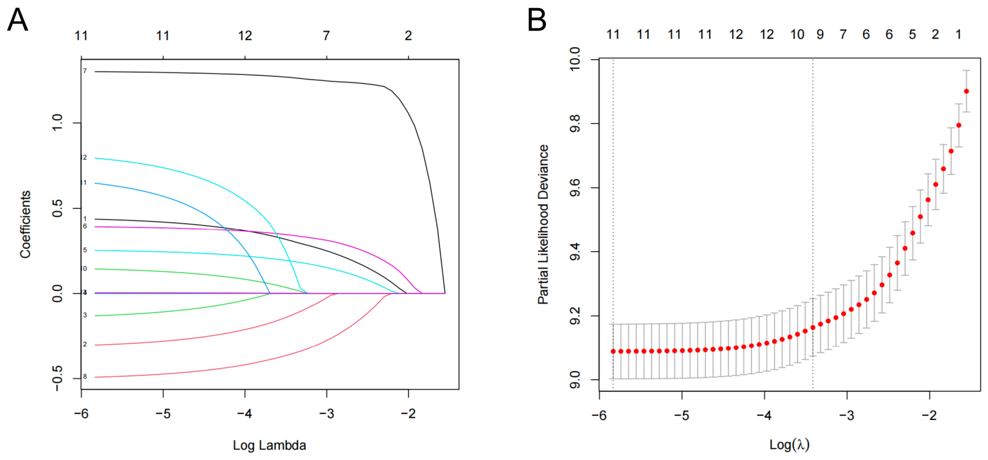

Figure S4. Predictor screening through the Least absolute shrinkage and selection operator (LASSO) regression model. (A) According to the logarithmic (lambda) sequence, a coefficient profile was generated, and non-zero coefficients were produced by the optimal lambda. (B) LASSO regression analysis determines the number of factors to build a model.

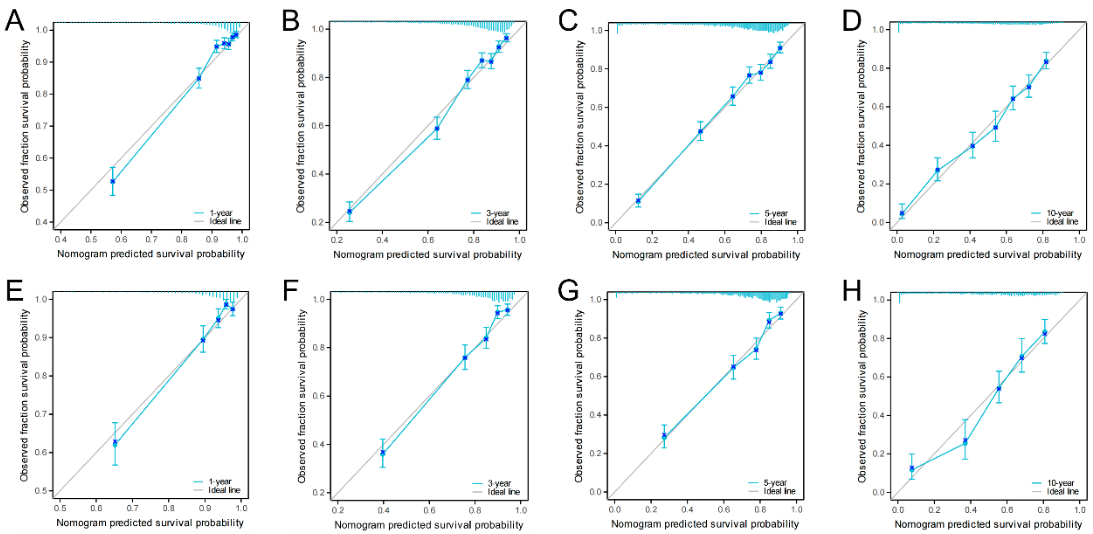

Figure S5. (A-D) The calibration curves of nomogram for predicting 1-, 3-, 5- and 10-year Overall survival (OS) of kidney cancer patients with second primary malignancy (SPM) in the training set. (E-H) The calibration curves of nomogram for predicting 1-, 3-, 5- and 10-year OS of kidney cancer patients with SPM in the testing set.

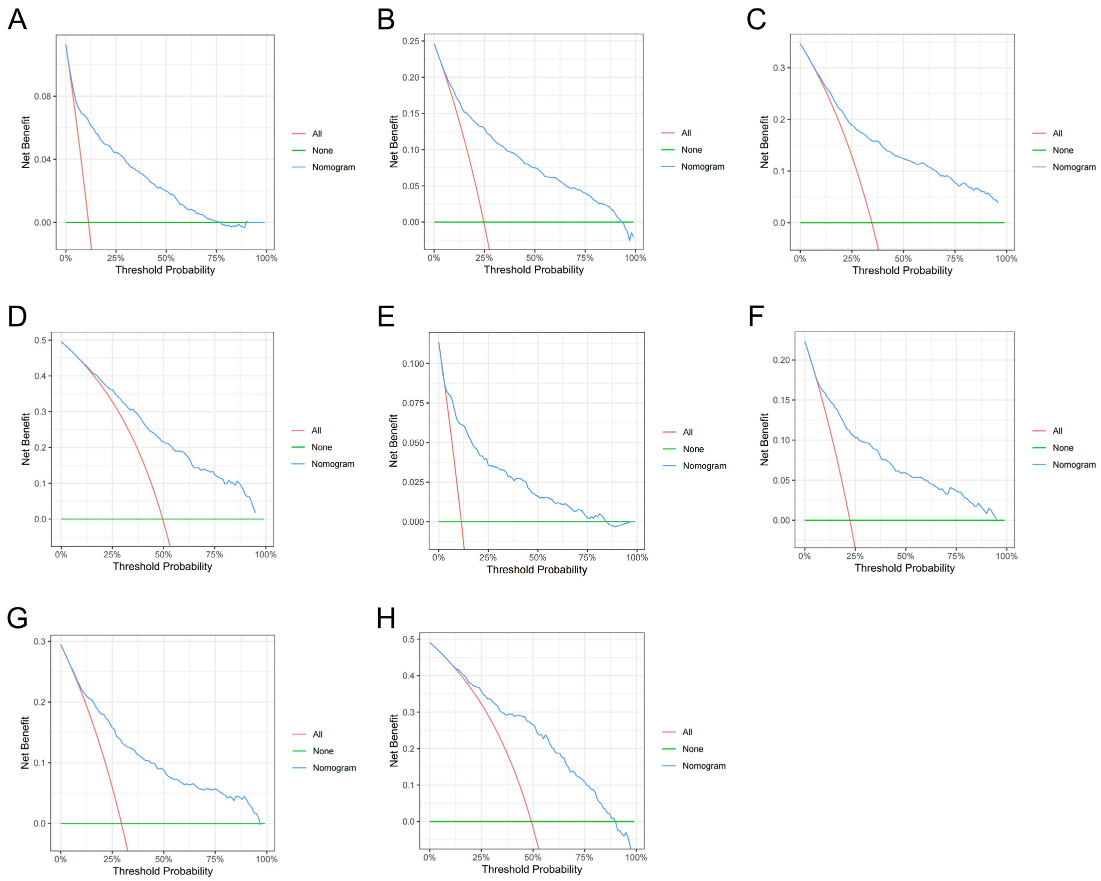

Figure S6. DCA curves for the training set (A-D) and testing set (E-H).

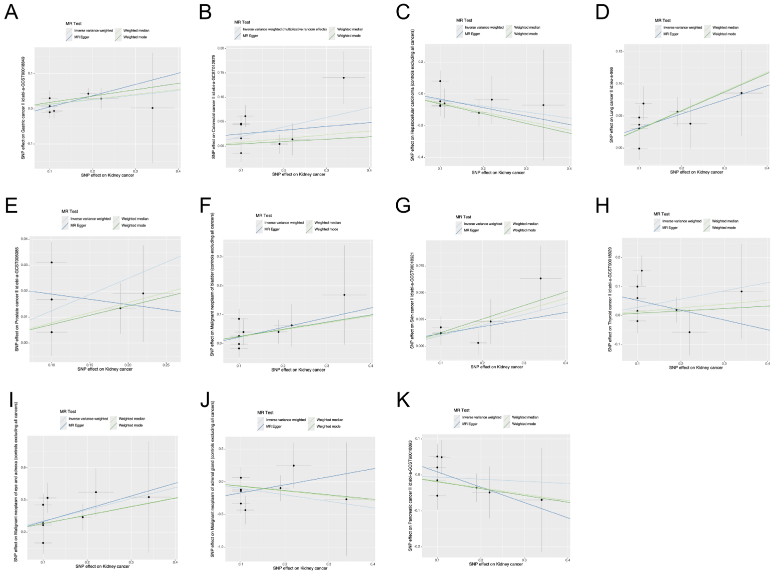

Figure S7. The scatter plots of Mendelian randomization analyses. (A) Kidney cancer on gastric cancer; (B) Kidney cancer on colorectal cancer; (C) Kidney cancer on hepatocellular carcinoma; (D) Kidney cancer on lung cancer; (E) Kidney cancer on prostate cancer; (F) Kidney cancer on bladder cancer; (G) Kidney cancer on skin cancer; (H) Kidney cancer on thyroid cancer; (I) Kidney cancer on eye and adnexa cancer; (J) Kidney cancer on adrenal gland cancer; (K) Kidney cancer on pancreatic cancer.

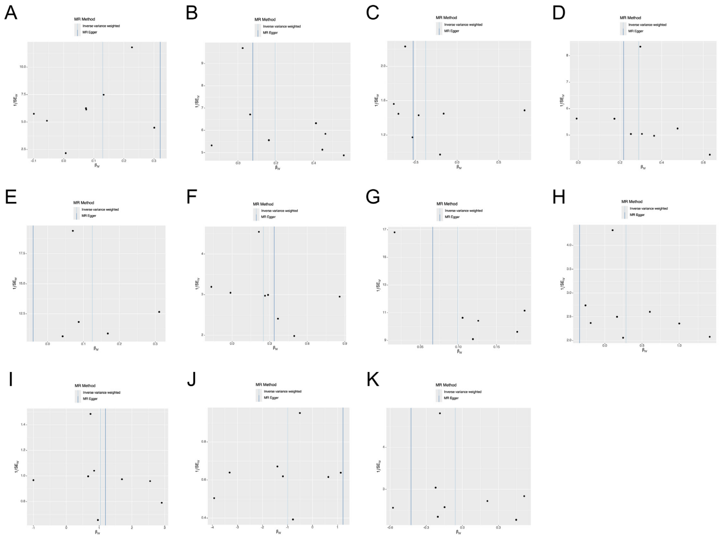

Figure S8. The funnel plots of Mendelian randomization analyses. (A) Kidney cancer on gastric cancer; (B) Kidney cancer on colorectal cancer; (C) Kidney cancer on

hepatocellular carcinoma; (D) Kidney cancer on lung cancer; (E) Kidney cancer on prostate cancer; (F) Kidney cancer on bladder cancer; (G) Kidney cancer on skin cancer; (H) Kidney cancer on thyroid cancer; (I) Kidney cancer on eye and adnexa cancer; (J) Kidney cancer on adrenal gland cancer; (K) Kidney cancer on pancreatic cancer.

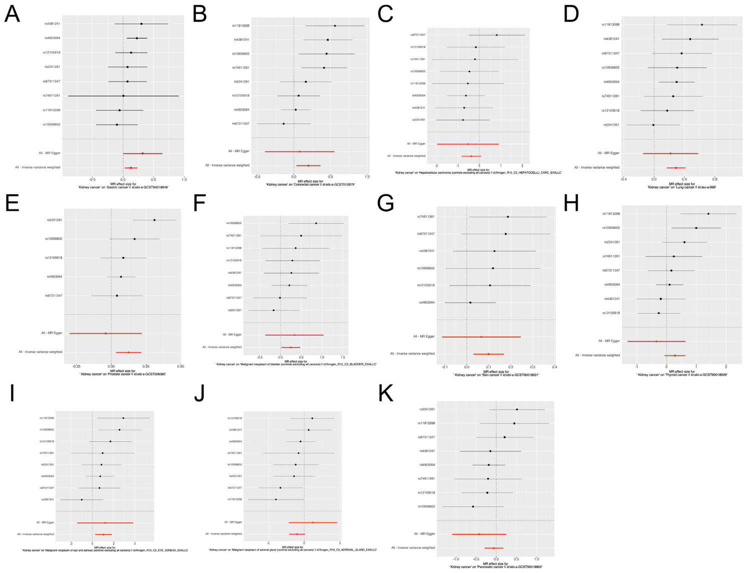

Figure S9. The forest plots of Mendelian randomization analyses. (A) Kidney cancer on gastric cancer; (B) Kidney cancer on colorectal cancer; (C) Kidney cancer on hepatocellular carcinoma; (D) Kidney cancer on lung cancer; (E) Kidney cancer on prostate cancer; (F) Kidney cancer on bladder cancer; (G) Kidney cancer on skin cancer; (H) Kidney cancer on thyroid cancer; (I) Kidney cancer on eye and adnexa cancer; (J) Kidney cancer on adrenal gland cancer; (K) Kidney cancer on pancreatic cancer.

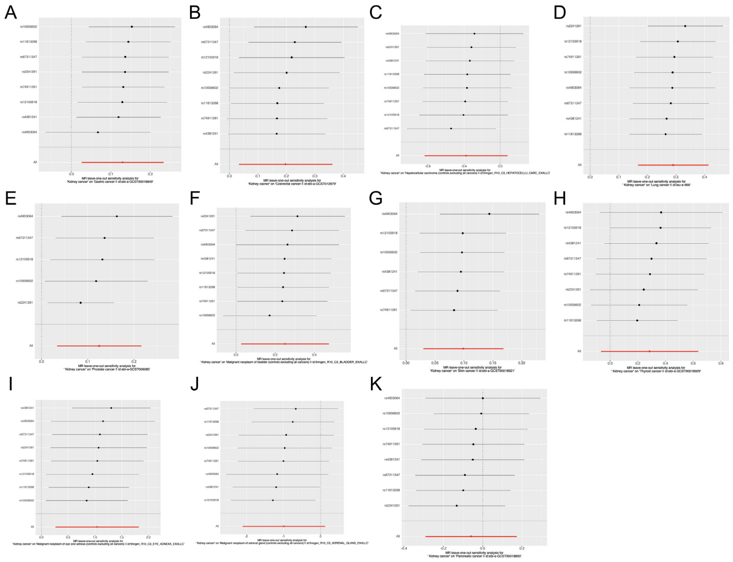

Figure S10. The leave-one-out plots of Mendelian randomization analyses. (A) Kidney cancer on gastric cancer; (B) Kidney cancer on colorectal cancer; (C) Kidney cancer on hepatocellular carcinoma; (D) Kidney cancer on lung cancer; (E) Kidney cancer on prostate cancer; (F) Kidney cancer on bladder cancer; (G) Kidney cancer on skin cancer; (H) Kidney cancer on thyroid cancer; (I) Kidney cancer on eye and adnexa cancer; (J) Kidney cancer on adrenal gland cancer; (K) Kidney cancer on pancreatic cancer.

Risk-based screen and prognostic analysis for second primary malignancies in kidney cancer patients: A retrospective cohort study based on large-scale population and mendelian randomization analysis

Supplementary Table S5-S6

Author names and affiliations: Mingrui Zou [1,2,3,†], Ruiyi Deng [1,2,3,†], Haode Liu [1,2,3,†], Jianhui Qiu [1,2,3], Peidong Tian [1,2,3], Jiaheng Shang [1,2,3], Jingcheng Zhou [1,2,3], Xueying Li[4], Lin Cai [1,2,3], Yizhou Wang [5,*] and Kan Gong [1,2,3,*]

[1]Department of Urology, Peking University First Hospital, Beijing, China.

[2]Institute of Urology, Peking University, Beijing, China.

[3]National Urological Cancer Center, Beijing, China.

[4]Department of Statistics, Peking University First Hospital, Beijing, China

[5]Department of Central Laboratory, Peking University First Hospital, Beijing, China.

[†]These authors contributed equally to this work and should be considered co-first authors.

* ***Corresponding authors***: Kan Gong(kan.gong@bjmu.edu.cn) AND Yizhou Wang (yizhouwang@bjmu.edu.cn)

Table S5. Demographic and clinical characteristics of first primary kidney cancer patients in the training set and testing set.

	Overall (n = 72408)	Training set (n = 50685)	Testing set (n = 21723)	P
**Age (%)**				0.841
≤ 50 years	17181 (23.7)	12007 (23.7)	5174 (23.8)	
50-60 years	20903 (28.9)	14608 (28.8)	6295 (29.0)	
60-70 years	21799 (30.1)	15263 (30.1)	6536 (30.1)	
70-80 years	12525 (17.3)	8807 (17.4)	3718 (17.1)	
**Sex (%)**				0.938
Female	27123 (37.5)	18991 (37.5)	8132 (37.4)	
Male	45285 (62.5)	31694 (62.5)	13591 (62.6)	
**Race (%)**				0.763
Black	7609 (10.5)	5334 (10.5)	2275 (10.5)	
White	59993 (82.9)	42009 (82.9)	17984 (82.8)	
Other	4806 (6.6)	3342 (6.6)	1464 (6.7)	
**Marital status (%)**				0.061
No/Divorced/Widowed/Unknown	26906 (37.2)	18722 (36.9)	8184 (37.7)	
Yes	45502 (62.8)	31963 (63.1)	13539 (62.3)	
**Income (%)**				0.010
≤ $75,000	47670 (65.8)	33520 (66.1)	14150 (65.1)	
> $75,000	24738 (34.2)	17165 (33.9)	7573 (34.9)	
**Rural/urban population density (%)**				0.330
Counties in metropolitan areas of over 1 million population	40539 (56.0)	28419 (56.1)	12120 (55.8)	
Counties in metropolitan areas of 0 to 1 millio n population	22086 (30.5)	15381 (30.3)	6705 (30.9)	

Nonmetropolitan counties	9783 (13.5)	6885 (13.6)	2898 (13.3)	
**Histologic type (%)**				0.286
ccRCC	47145 (65.1)	32875 (64.9)	14270 (65.7)	
chRCC	3441 (4.8)	2434 (4.8)	1007 (4.6)	
Other	13038 (18.0)	9204 (18.2)	3834 (17.6)	
pRCC	8429 (11.6)	5924 (11.7)	2505 (11.5)	
sRCC	355 (0.5)	248 (0.5)	107 (0.5)	
**Grade (%)**				0.891
I	9837 (13.6)	6906 (13.6)	2931 (13.5)	
II	39542 (54.6)	27664 (54.6)	11878 (54.7)	
III/IV	23029 (31.8)	16115 (31.8)	6914 (31.8)	
**T stage (%)**				0.858
T1	52256 (72.2)	36554 (72.1)	15702 (72.3)	
T2	8084 (11.2)	5679 (11.2)	2405 (11.1)	
T3	12068 (16.7)	8452 (16.7)	3616 (16.6)	
**Surgery(%)**				0.646
Not performed	642 (0.9)	439 (0.9)	203 (0.9)	
PN	25165 (34.8)	17591 (34.7)	7574 (34.9)	
RN	44186 (61.0)	30966 (61.1)	13220 (60.9)	
LTE	868 (1.2)	620 (1.2)	248 (1.1)	
Other	1547 (2.1)	1069 (2.1)	478 (2.2)	
**Laterality (%)**				0.266
Bilateral	43 (0.1)	34 (0.1)	9 (0.0)	
Left	35331 (48.8)	24790 (48.9)	10541 (48.5)	
Right	37034 (51.1)	25861 (51.0)	11173 (51.4)	
**Radiotherapy (%)**				0.912
No/Unknown	72157 (99.7)	50508 (99.7)	21649 (99.7)	
Yes	251 (0.3)	177 (0.3)	74 (0.3)	

**Chemotherapy (%)**				0.458
No/Unknown	71497 (98.7)	50058 (98.8)	21439 (98.7)	
Yes	911 (1.3)	627 (1.2)	284 (1.3)	

Table S6. Demographic and clinical characteristics of first primary kidney cancer patients with SPM in the training set and testing set.

	Overall (n = 5295)	Training set (n= 3706)	Testing set (n= 1589)	P
**Age (%)**				0.981
≤ 50 years	606 (11.4)	428 (11.5)	178 (11.2)	
50-60 years	1503 (28.4)	1053 (28.4)	450 (28.3)	
60-70 years	2122 (40.1)	1480 (39.9)	642 (40.4)	
70-80 years	1064 (20.1)	745 (20.1)	319 (20.1)	
**Sex (%)**				0.470
Female	1769 (33.4)	1250 (33.7)	519 (32.7)	
Male	3526 (66.6)	2456 (66.3)	1070 (67.3)	
**Race (%)**				0.703
Black	606 (11.4)	426 (11.5)	180 (11.3)	
White	4385 (82.8)	3061 (82.6)	1324 (83.3)	
Other	304 (5.7)	219 (5.9)	85 (5.3)	
**Marital status (%)**				0.252
No/Divorced/Widowed/Unknown	2059 (38.9)	1422 (38.4)	637 (40.1)	
Yes	3236 (61.1)	2284 (61.6)	952 (59.9)	
**Income (%)**				0.654
≤ $75,000	3223 (60.9)	2248 (60.7)	975 (61.4)	
> $75,000	2072 (39.1)	1458 (39.3)	614 (38.6)	
**Rural/urban population density (%)**				0.360
Counties in metropolitan areas of over 1 million population	2953 (55.8)	2084 (56.2)	869 (54.7)	

Counties in metropolitan areas of 0 to 1 milli on population	1649 (31.1)	1152 (31.1)	497 (31.3)	
Nonmetropolitan counties	693 (13.1)	470 (12.7)	223 (14.0)	
**Interval between diagnoses (Mean (SD))**				0.331
Interval	56.54 (40.54)	56.19 (40.33)	57.37 (41.01)	
**Site of SPM (%)**				0.620
Urinary system	1761 (33.3)	1215 (32.8)	546 (34.4)	
Digestive system	944 (17.8)	657 (17.7)	287 (18.1)	
Reproductive system	938 (17.7)	674 (18.2)	264 (16.6)	
Respiratory system	838 (15.8)	592 (16.0)	246 (15.5)	
Other	814 (15.4)	568 (15.3)	246 (15.5)	
**T stage of SPM (%)**				0.153
T1	2587 (48.9)	1844 (49.8)	743 (46.8)	
T2	1535 (29.0)	1056 (28.5)	479 (30.1)	
T3	808 (15.3)	547 (14.8)	261 (16.4)	
T4	365 (6.9)	259 (7.0)	106 (6.7)	
**N stage of SPM (%)**				0.666
N0	4250 (80.3)	2970 (80.1)	1280 (80.6)	
N1	625 (11.8)	448 (12.1)	177 (11.1)	
N2	325 (6.1)	225 (6.1)	100 (6.3)	
N3	95 (1.8)	63 (1.7)	32 (2.0)	
**M stage of SPM (%)**				0.239
M0	4854 (91.7)	3386 (91.4)	1468 (92.4)	
M1	441 (8.3)	320 (8.6)	121 (7.6)	
**Surgery status of SPM (%)**				0.519
Not performed	1785 (33.7)	1260 (34.0)	525 (33.0)	
Surgery performed	3510 (66.3)	2446 (66.0)	1064 (67.0)	

**Radiotherapy of SPM (%)**				1.000
No/Unknown	3566 (67.3)	2496 (67.4)	1070 (67.3)	
Yes	1729 (32.7)	1210 (32.6)	519 (32.7)	
**Chemotherapy of SPM (%)**				0.149
No/Unknown	4185 (79.0)	2909 (78.5)	1276 (80.3)	
Yes	1110 (21.0)	797 (21.5)	313 (19.7)	
**Histologic type of FPM (%)**				0.436
ccRCC	3253 (61.4)	2250 (60.7)	1003 (63.1)	
chRCC	240 (4.5)	175 (4.7)	65 (4.1)	
Other	1029 (19.4)	736 (19.9)	293 (18.4)	
pRCC	754 (14.2)	533 (14.4)	221 (13.9)	
sRCC	19 (0.4)	12 (0.3)	7 (0.4)	
**Grade of FPM (%)**				0.697
I	776 (14.7)	556 (15.0)	220 (13.8)	
II	2913 (55.0)	2024 (54.6)	889 (55.9)	
III	1377 (26.0)	964 (26.0)	413 (26.0)	
IV	229 (4.3)	162 (4.4)	67 (4.2)	
**Laterality of FPM (%)**				0.619
Bilateral	2 (0.0)	2 (0.1)	0 (0.0)	
Left	2637 (49.8)	1840 (49.6)	797 (50.2)	
Right	2656 (50.2)	1864 (50.3)	792 (49.8)	
**Surgery status of FPM (%)**				0.162
Not performed	30 (0.6)	25 (0.7)	5 (0.3)	
Surgery performed	5265 (99.4)	3681 (99.3)	1584 (99.7)	
**Radiotherapy of FPM (%)**				0.388
No/Unknown	5264 (99.4)	3687 (99.5)	1577 (99.2)	
Yes	31 (0.6)	19 (0.5)	12 (0.8)	
**Chemotherapy of FPM (%)**				0.352

No/Unknown	5219 (98.6)	3657 (98.7)	1562 (98.3)	
Yes	76 (1.4)	49 (1.3)	27 (1.7)	
**Size of FPM (%)**				0.529
≤ 2 cm	673 (12.7)	459 (12.4)	214 (13.5)	
2-3 cm	1025 (19.4)	726 (19.6)	299 (18.8)	
3-4 cm	979 (18.5)	675 (18.2)	304 (19.1)	
> 4cm	2618 (49.4)	1846 (49.8)	772 (48.6)	
